# Inferior Hepatic Fissures: Anatomic Variants in Trinidad and Tobago

**DOI:** 10.7759/cureus.8369

**Published:** 2020-05-30

**Authors:** Shamir O Cawich, Michael T Gardner, Mickhaiel Barrow, Shaheeba Barrow, Dexter Thomas, Vindra Ragoonanan, Avidesh Mahabir, Reyad Ali, Vijay Naraynsingh

**Affiliations:** 1 Surgery, University of the West Indies, St. Augustine, TTO; 2 Anatomy, University of the West Indies, Kingston, JAM; 3 Pathology, Port of Spain General Hospital, Port of Spain, TTO; 4 Surgery, Port of Spain General Hosptial, Port of Spain, TTO; 5 Surgery, Port of Spain General Hospital, Port of Spain, TTO; 6 Pathology, Port of Spain General Hosptial, Port of Spain, TTO; 7 Surgery, Medical Associates Hospital, St. Joseph, TTO; 8 Clinical Surgical Sciences, University of the West Indies, St. Augustine, TTO

**Keywords:** liver, fissure, hepatic, variant, anatomy, caribbean, trinidad

## Abstract

Classic descriptions of the visceral surface of the human liver only define three fissures: transverse, sagittal and umbilical fissures. Any additional fissures that are present on the visceral surface of the liver are considered variant inferior hepatic fissures (IHFs). This study was carried out to document the prevalence of IHFs in the Eastern Caribbean. Knowledge of these variants is important to clinicians who treat liver disorders in persons of the Caribbean diaspora.

In this study, two independent researchers observed all consecutive autopsies performed at the facility over a period of 10 weeks. They examined the visceral surface of the unfixed liver in situ. Any specimen with variant IHFs was selected for detailed study. We documented the relation of the variant IHFs to nearby viscera and then explanted the livers using a standardized technique. The following details were recorded for each liver: number, location, depth, length, and width of IHFs. All measurements were checked independently by two researchers and the average measurement was used as the final dimension. Each liver was then sectioned in 1 cm sagittal slices to document the relationship of intraparenchymal structures.

We observed 60 consecutive autopsies in unselected cadavers. Variant IHFs were present in 21 (35%) cadavers at a mean age of 68.25 years (range: 61 - 83; median 64.5; standard deviation (SD) ± 8.45). The variants included a deep fissure in the coronal plane between segments V and VI in 19 (31.7%) cadavers (related to the right branch of the portal vein in 63.2% of cases), a well-defined segment VI fissure running in a sagittal plane in four (6.7%) cadavers, a well-defined fissure incompletely separating the caudate process from the caudate lobe proper in five (8.3%) cadavers, a consistent fissure that arose from the left side of the transverse fissure and coursed between segments II and III in three (5%) cadavers, and a deep coronal fissure dividing the quadrate to form an accessory quadrate lobe in one (1.7%) cadaver.

Almost one in three unselected persons in this population have anatomically variant fissures on the visceral surface of the liver. The variants include Rouvière’s sulci (31.7%), caudate notches (8.3%), segment VI fissures (6.7%), left medial segment fissures (5%), and quadrate fissures (1.7%). The clinical relevance of these variants is discussed. Any clinician treating liver diseases in persons of Caribbean extract should be aware of their presence.

## Introduction

There are many documented variations of human liver morphology. Classically, there are only three fissures on the visceral surface and any additional fissure is termed a variant inferior hepatic fissure (IHF). This study was carried out to document the presence of variant IHFs in Trinidad and Tobago. This is the most populous island in the Eastern Caribbean with a population of 1.35 million persons and equal proportions of persons of Afro-Caribbean and Indio-Caribbean descent. It is important for clinicians who treat liver disorders in persons from the Caribbean diaspora to be aware of the existing variations.

## Materials and methods

This study was performed in the Pathology Department at the Port of Spain General Hospital. This facility is a tertiary referral hospital servicing a catchment population of 650,000 persons in the northwestern part of Trinidad and Tobago. This facility is the main referral centre for pathology services for the public healthcare system of Trinidad and Tobago. After securing approval from the institutional review board, we performed an observational study during autopsies at this facility.

**Figure 1:**
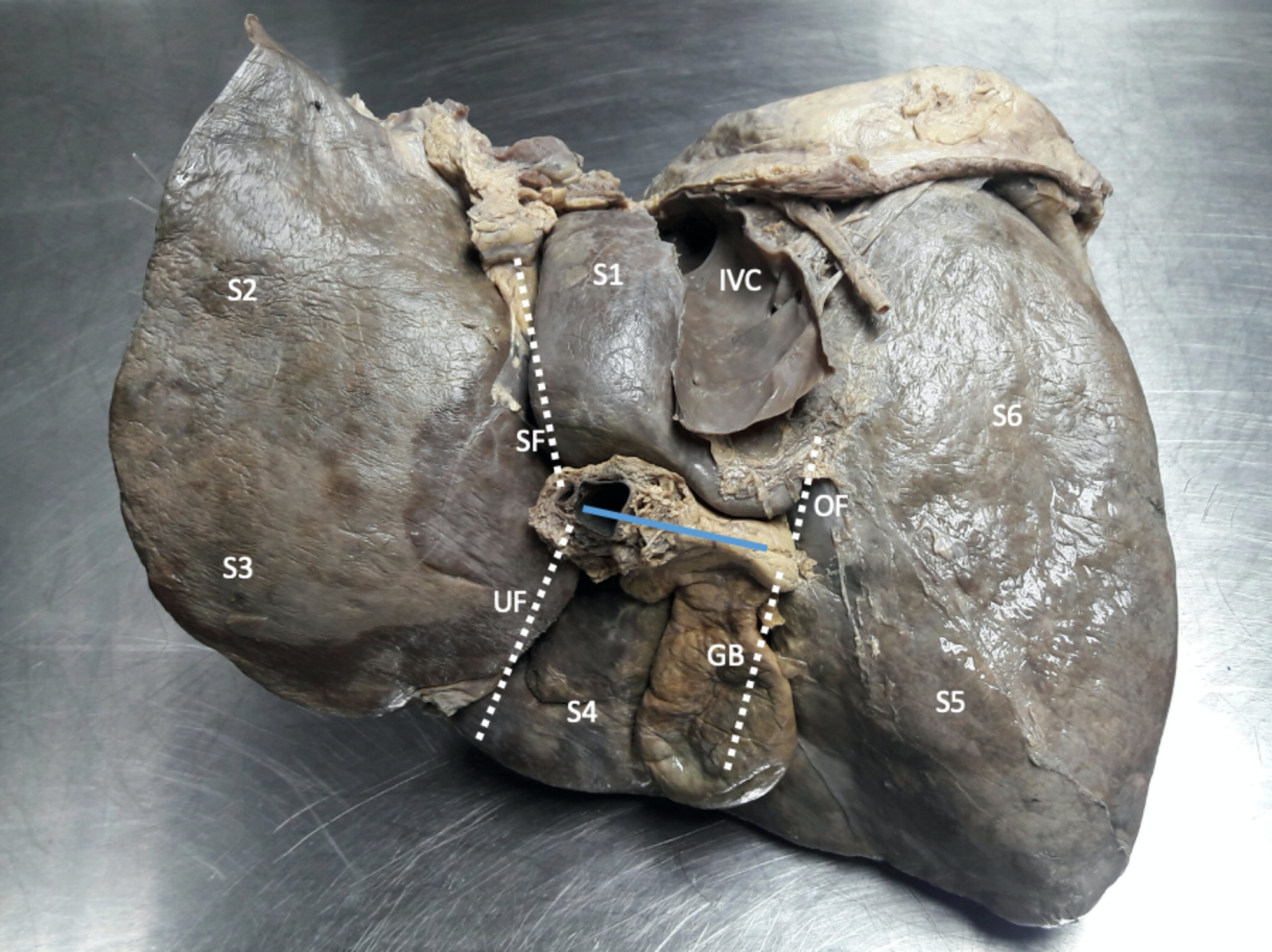
A view of the visceral surface of an explanted cadaveric liver illustrating classic anatomy The visceral surface is divided into four areas, roughly in a pattern resembling the letter “H”. The transverse fissure (solid line) forms the central stem of the “H” and runs in a coronal plane, separating the caudate (S1) and quadrate (S4) lobes. At the left end of the transverse fissure, the sagittal fissure (SF) and umbilical fissure (UF) course in opposite directions to separate the left medial (S4) and left lateral (S2 and 3) sections. The oblique fissure (OF) runs from the right side of the transverse fissure toward the inferior vena cava (IVC). An imaginary line connecting the middle of the gallbladder (GB) fossa and the oblique fissure separates the left and right hemiliver.

The classic descriptions of the visceral surface of the liver were used to define “normal” anatomy [1-3]. Figure 1 illustrates the classic description of the visceral liver surface, where four areas, roughly resembling the letter “H”, are delineated by the gallbladder fossa, inferior vena cava (IVC), transverse, sagittal, and umbilical fissures. The transverse fissure forms the central stem of the “H” and runs in a coronal plane, separating the caudate (posteriorly) and quadrate (anteriorly) lobes. This is the point at which the hepatic portal triad enters the liver.

The caudate lobe (segment 1) is outlined by the transverse fissure anteriorly, the sagittal fissure at its left border, the oblique fissure on its right border, and the IVC posteriorly (Figure 1). The oblique fissure extends from the gallbladder fossa to the IVC posteriorly. Cantlie’s plane runs along the oblique fissure and gallbladder fossa to separate the left and right hemiliver. 

On the left side of the transverse fissure, the sagittal and umbilical fissures course in opposite directions, separating the medial and lateral sections of the left hemiliver. The sagittal fissure (aka fissure for the ligamentum venosum) forms the left border of the caudate lobe (segment 1) and extends anteriorly to join the transverse fissure. The umbilical fissure (aka fissure for ligamentum teres) extends from the transverse fissure to the anterior liver edge, separating segments 3 and 4b along its path.

There are no other IHFs described in classic anatomic descriptions of the liver [1-3]. Any additional fissures encountered in our study were considered anatomic variants. In this study, two independent researchers observed all consecutive autopsies performed at the facility over a period of 10 weeks. They examined the visceral surface of the unfixed liver in situ. Any specimen with variant IHFs was selected for detailed study. We documented the relation of the variant IHFs to nearby viscera. The livers were then explanted by interrupting the triangular and coronary ligaments, transecting the hepatoduodenal ligament and the IVC 2 cm away from the liver's edge. The following details were recorded for each liver: number, location, depth, length, and width of the IHFs. All measurements were taken with a standardized metal ruler and checked independently by each of the two researchers. The average measurement was used as the final dimension. Each liver was then sectioned in 1 cm sagittal slices to document the relationship of intraparenchymal structures.

## Results

Over the study period, we observed 60 autopsies in unselected consecutive cadavers. We encountered variant IHFs in 21 (35%) cadavers (Table 1). The mean age of the cadavers with variant IHFs was 68.25 years (range: 61 - 83; median 64.5; SD ± 8.45).

**Table 1:** Anatomic Details of Variant Inferior Hepatic Sulci in 60 Specimens

Variant Fissure	No	Percent	Length (cm)	Width (cm)	Depth (cm)	Special observations
Rouvière’s sulcus	19	31.7%	7.1	1.4	1.2	Right vascular pedicle found at the base
Caudate notch	5	8.3%	1.0	0.8	0.7	Associated with caudate process
Segment 6 fissure	4	6.7%	5.1	0.9	1.0	None
Left medial segment fissure	3	5.0%	1.2	1.1	1.0	Associated with left lingular process
Quadrate fissure	1	1.7%	0.6	0.3	0.4	Associated with accessory lobe of the quadrate

**Figure 2:**
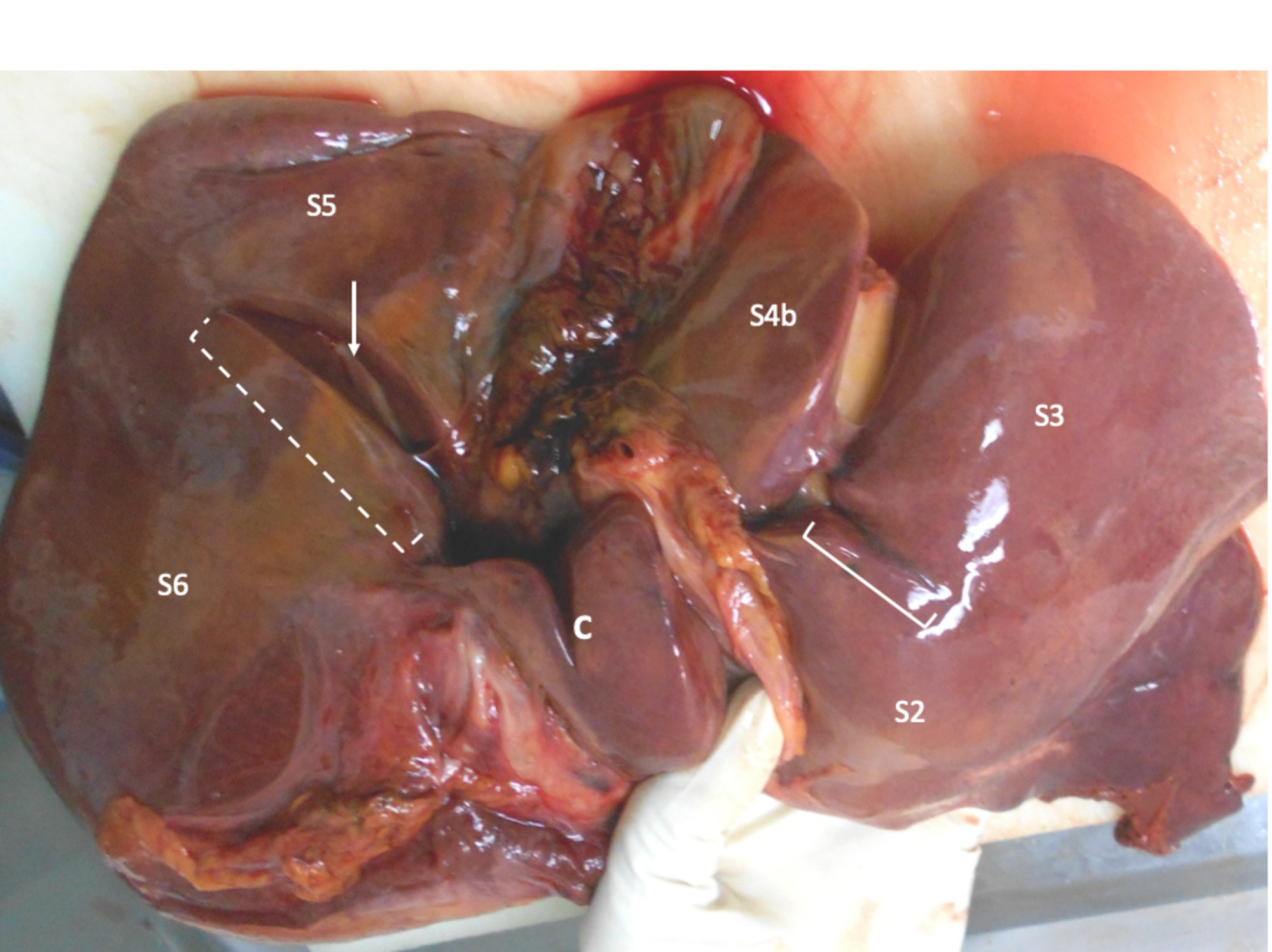
Fissure between segments 5 and 6 Multiple variants are visible on the visceral surface of this liver. There is an open-type Rouvière’s sulcus (broken line) separating segments 5 (S5) and 6 (S6). The right branch of the portal vein (arrow) is visible at the floor of the fissure covered by a thin layer of Glisson’s capsule. There is also a caudate notch (C) present that incompletely divides the caudate lobe in the sagittal plane. A third fissure (solid line) extends from the left side of the transverse fissure and continues into the left lateral section to separate segments 2 (S2) and 3 (S3).

Most of the variants were present at the right hemiliver. The commonest variant was a deep fissure lying in a coronal plane between segments 5 and 6 that was encountered in 19 (31.7%) cadavers (Figure 2). This IHF commenced at the lateral extent of the transverse fissure, near the gallbladder infundibulum. The fissure extended laterally for variable distances into the right liver, usually extending toward the upper pole of the right kidney. The right branch of portal vein could be found at the floor of the sulcus in all cases. In 12 (63.2%) of the cases, the portal vein at the fissure floor was covered only by a thin layer of Glisson’s capsule, and in the remainder, there was a bridge of parenchyma partially covering the floor of the fissure.

**Figure 3:**
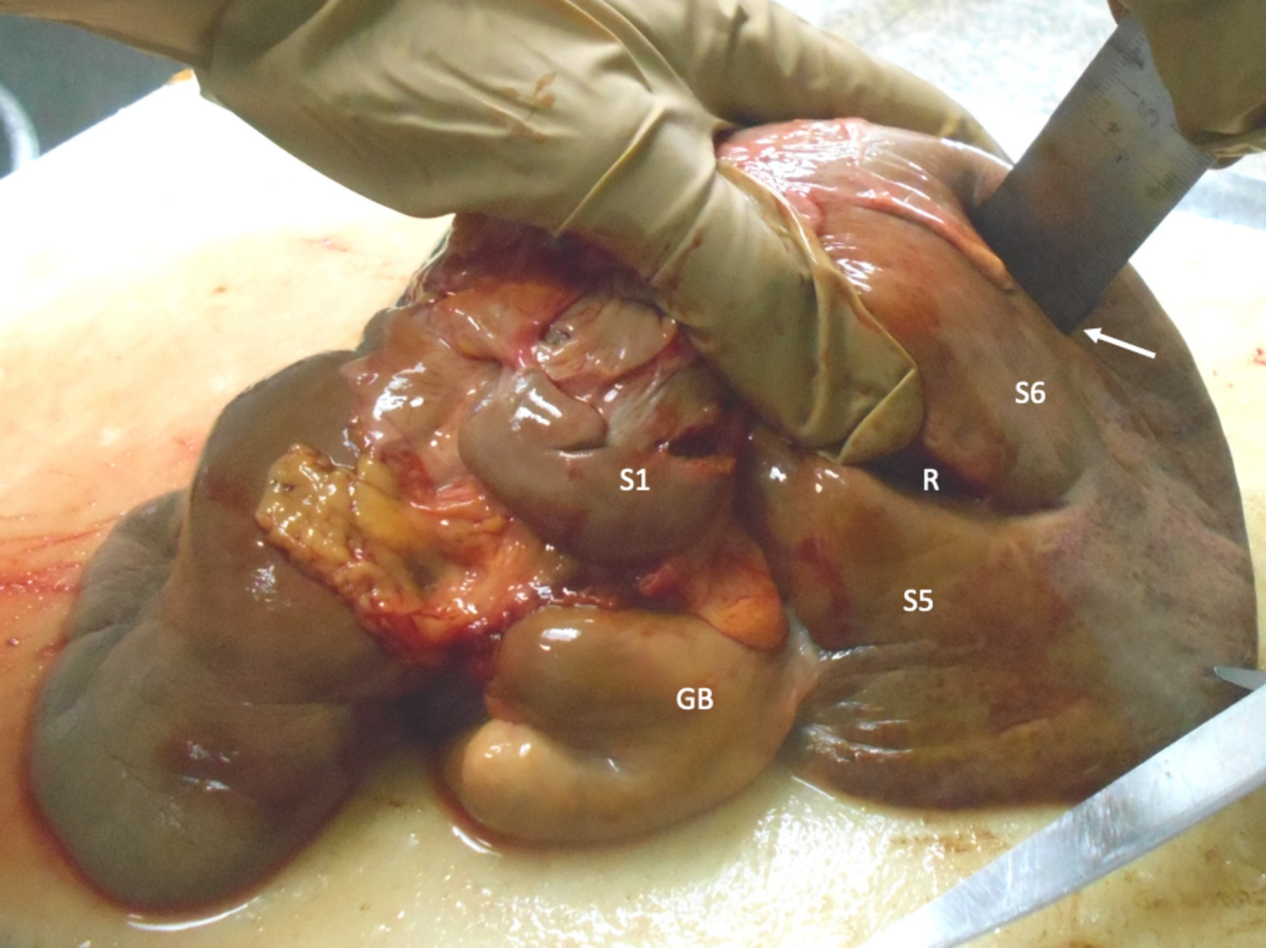
Segment 6 fissure This specimen demonstrates an open-type Rouvière’s sulcus (R) separating segments 5 (S5) and 6 (S6). There is also a well-developed fissure in segment 6 roughly oriented in a sagittal plane (arrow), as well as a bilobed gallbladder (GB). The caudate lobe (S1) is labeled for orientation.

The second variant encountered in the right hemiliver was a well-defined fissure at segment 6 running in a sagittal plane that was seen in four (6.7%) cadavers (Figure 3). The colic, renal, and duodenal impressions in these cadavers appeared normal. On the in-situ examination, there were no abnormalities at adjacent viscera in any of the specimens. There was no apparent relationship between this IHF and any vascular structure on sectioning.

The caudate lobe was the second most common segment to harbor variant IHF. Five (8.3%) cadavers had a well-defined fissure incompletely separating the caudate process from the caudate lobe proper on the visceral surface of the liver (Figure 2). No vascular structures were seen in immediate relation to this fissure on sectioning. 

**Figure 4:**
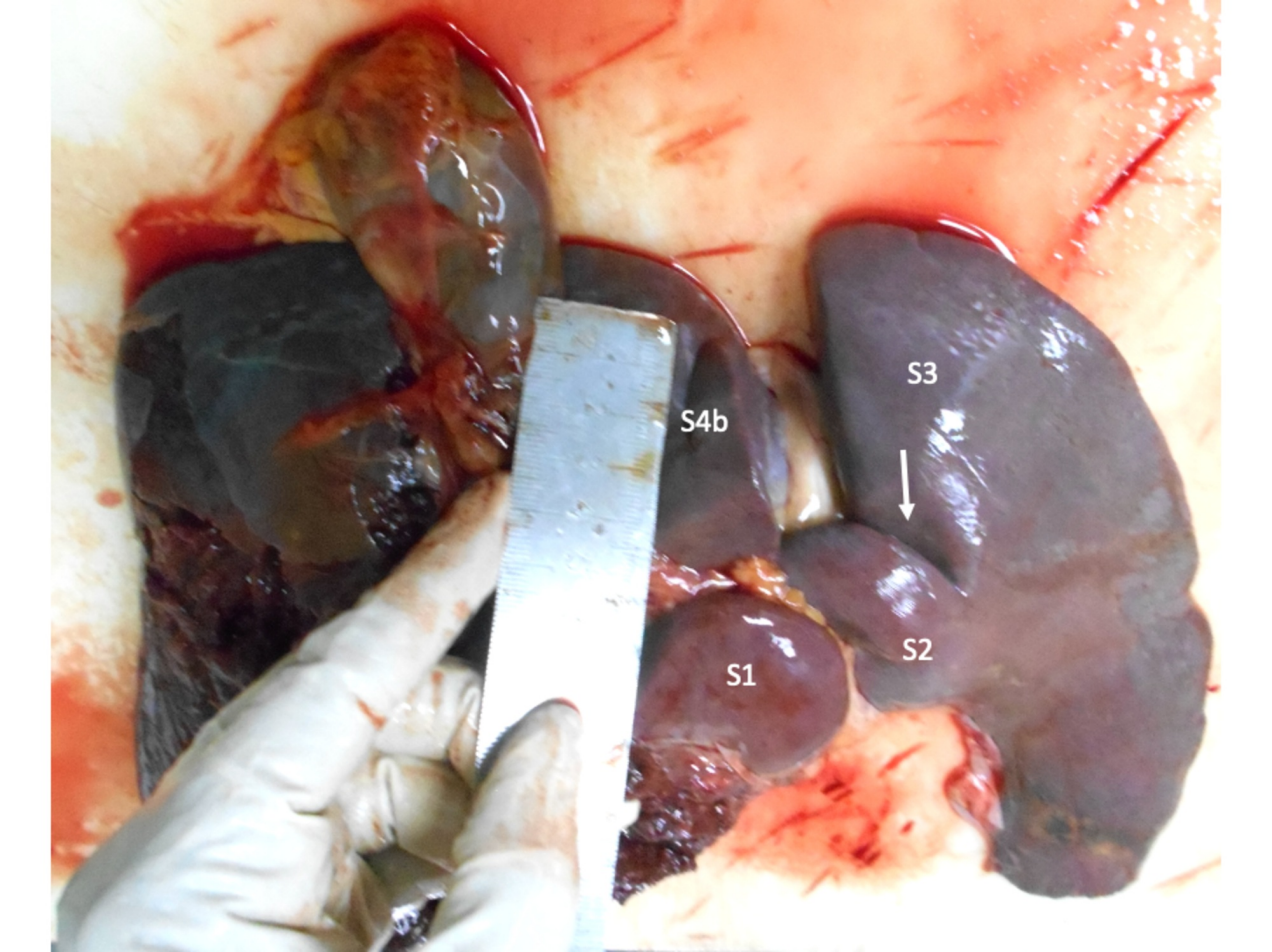
Fissure between segments 2 and 3 Visceral surface of a liver demonstrating a well-defined fissure (arrow) that extends from the left end of the transverse fissure and courses into the left lateral section to separate segment 2 (S2) and segment 3 (S3). The caudate (S1) and quadrate (S4b) lobes are labeled for orientation.

Three (5%) cadavers had a consistent fissure in the left hemiliver that arose from the left side of the transverse fissure and coursed between segments 2 and 3 for varying distances (Figure 4). These three cadavers also had a lingular process of the left lobe.

**Figure 5:**
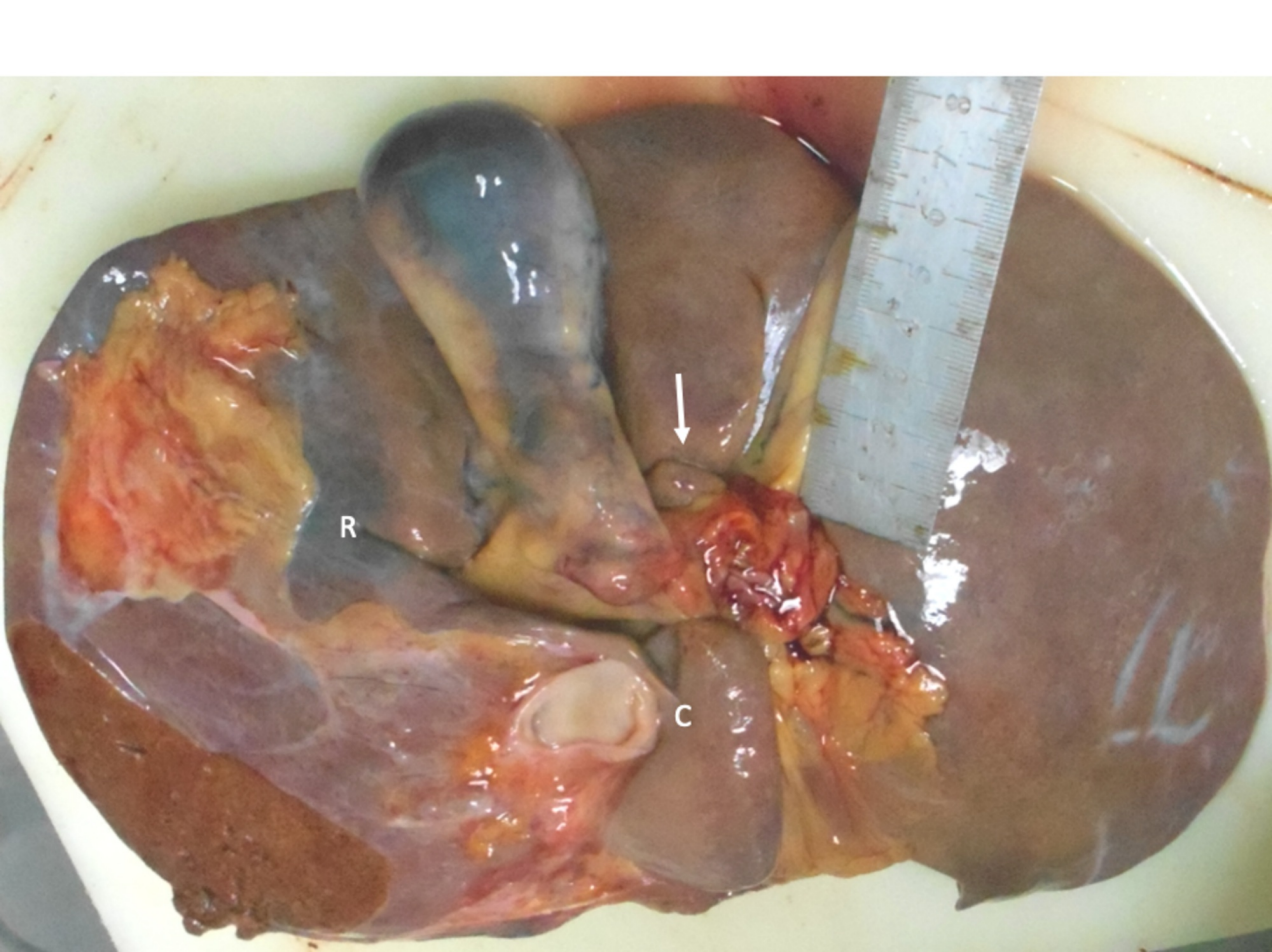
Quadrate lobe fissure Multiple variant fissures are present on the visceral surface of this liver, including an open-type Rouvière’s sulcus (R), a caudate notch (C), and a fissure in the quadrate lobe (arrow) that runs in a coronal plane to create an accessory lobe.

The least common area to encounter variant IHF was the quadrate lobe. Only one (1.7%) cadaver had a deep fissure running in a coronal plane, dividing the quadrate to form an accessory quadrate lobe (Figure 5).

There were a few associated morphologic abnormalities observed in the specimens with IHFs. These included left lingular processes (3), bi-lobed gallbladder (1), and the accessory lobe of the quadrate lobe (1). 

## Discussion

It is important for clinicians to be aware of the presence of these IHF variants. Radiologists who are unaware of their presence may mistake them for pathologic lesions, such as liver metastases, abscesses, or haematomas [3-5]. Hepatobiliary surgeons may also use them as landmarks to plan liver resections and may modify operative techniques based on their presence [6-7].

There is a wide range in the incidence of IHF variants reported in the medical literature [8-16], ranging from a low of 0.8% [4] and up to 82% [8]. The incidence in our population (35%) fell roughly within this range without a gender predilection. 

The commonest variant we encountered was a deep IHF that extended from the right side of the transverse fissure, along the right intersectional plane. These fissures have been described before in the medical literature, but there has been an inconsistency in nomenclature. Similar fissures have been named the “inferior accessory hepatic fissure” by Lim et al., “incisura dextra of Gans” by Reynaud et al., and “le sillon du processus caude” by Rouvière [9-11]. In modern medical literature, it is most often called “Rouvière’s sulcus” in credit to Henri Rouvière who first described it in 1924 [11]. Its prevalence ranges across the globe from 11% in India to 82% in Slovenia [8, 12]. The prevalence of Rouvière’s sulcus in our population (31.7%) was most closely related to that in North India (28%) as reported by Joshi et al. [4]. This was interesting since a large proportion of the population in Trinidad and Tobago is from the East Indian diaspora. The prevalence of Rouvière’s sulcus was greater than in a report from the Northern Caribbean where it was noted in 12% of persons [16]. This report originated in Jamaica where the majority of persons sampled were of Afro-Caribbean ethnicity [16].

In 63.2% of our cases, Rouvière’s sulcus was continuous with the transverse fissure and only covered by a thin layer of Glisson’s capsule, allowing the right branch of the portal vein to be visualized. Zubair et al. described this as an open-type sulcus, in contrast to the fused-type where a parenchymal bridge interrupted the fissure, so it was only visible at the lateral end [13]. Our findings were comparable to those in the medical literature, where the open-type fissures were reported in 45% to 85% of persons with a Rouvière’s sulcus [8, 13].

In all open-type fissures in our study, the right branch of the portal vein could be seen at the fissure floor. In the remaining seven cases with a closed-type, dissection of the parenchymal bridge revealed a similar relationship with the right portal structures. This was expected as many authors have reported a high correlation between Rouvière’s sulcus and the right portal triad on cadaveric dissections and in radiologic studies [3-4, 8-9, 15]. For this reason, surgeons use it as a landmark when performing laparoscopic liver resections and cholecystectomies [8, 13, 17]. 

The caudate notch was the second most common variant IHF we encountered. Sagoo et al. defined this as a fissure on the visceral surface of the liver that separates the normally cuboid bridge of the caudate parenchyma into the caudate lobe proper and a caudate process [18]. We only found this in 8.3% of our population, but the worldwide prevalence varies from 9% in Northern India to 100% at Karnataka in Southwest India [19-20]. Kogure et al. reported that an underlying vein correlated to the presence of the caudate notch, but this relationship was not observed in our population [21].

The third most common variant we encountered was an IHF at segment 6 oriented in a coronal plane. We could not find a relationship between this fissure and the underlying biliary radicles, vascular structures, liver parenchymal diseases, or intra-abdominal viscera. Othman suggested that this fissure was due to “pressure exerted by the colon” [14]. However, in our four cases, the ligamentous attachments were quite lax, leaving little contact between the hepatic flexure of the colon and segment 6. Therefore, at least in our cases, it seems unlikely that this fissure resulted from compression by the colon.

The fourth most common variant in our population was a deep fissure coursing in a sagittal plane into the left lateral section between segments 2 and 3. It was only present in 5% of cadavers, but the second-order left hepatic pedicle was always found on the floor of this fissure. Therefore, when present, it can be an important landmark for hepatobiliary surgeons performing a left lateral sectionectomy. 

The quadrate fissure was the least common IHF variant in our study. This was not unusual because there are very few reports in the literature. Nayak published an image of a similar fissure and accessory caudate lobe found in 1 (1.8%) of 55 livers and Baruah et al. published a similar image found in 1 (3.3%) of 30 livers in their study [22-23]. Their clinical significance is uncertain as there have been no reports demonstrating any relationship with underlying anatomical structures or any clinical sequelae. 

## Conclusions

Almost one in three unselected persons in this Trinidadian population have anatomically variant fissures on the visceral surface of the liver. The variants include Rouvière’s sulci (31.7%), caudate notches (8.3%), segment 6 fissures (6.7%), left medial segment fissures (5%), and quadrate fissures (1.7%). These variants have clinical relevance, and any clinician treating liver diseases in persons of Caribbean extract should be aware of their presence. 
